# Mechanism of resistance to immune checkpoint inhibitors

**DOI:** 10.20517/cdr.2018.015

**Published:** 2019-06-19

**Authors:** Manu R. Pandey, Marc S. Ernstoff

**Affiliations:** Department of Medicine, Roswell Park Comprehensive Cancer Center, NY 14203, USA.

**Keywords:** Immune checkpoint inhibitors, checkpoint inhibitors, resistance, mechanism

## Abstract

Immune checkpoint inhibitors (ICI) have revolutionized the management of cancer over the last decade. Instead of targeting the cancer cell directly these agents work by augmenting the immune response towards tumor. Although they are associated with improved responses compared to traditional treatments in several malignancies, a majority of the patients don't respond to ICIs even when used in the frontline setting. In patients who do respond, a significant number eventually develop resistance. We will review ICI mechanisms of action and resistance. We will also discuss new therapeutic options and combinations with other agents that are currently being evaluated to overcome resistance to ICI.

## Introduction

Several immune checkpoint inhibitors (ICI) have been approved for use in metastatic cancers as well as in the adjuvant setting. Ipilimumab, an anti-cytotoxic T-lymphocyte associated protein-4 (CTLA-4) monoclonal antibody (mab) was the first ICI to be approved for use in metastatic melanoma. This was followed by anti-programmed cell death-1 (PD-1) mabs pembrolizumab and nivolumab that were initially approved for melanoma as well, but have since also received approval for several other cancers. The latest ICI to receive approval are anti-programmed death-ligand1 (PD-L1) mabs which include avelumab, druvalumab and atezolizumab.

Although immune checkpoint inhibitors have brought a paradigm shift in the management of several cancers, many patients will have primary resistance to treatment. Ipilimumab, an anti-CTLA4 monoclonal antibody can elicit an objective response in 10%-15% of metastatic melanoma patients^[1,2]^. Even in responders, 40% of the patients had progression within 2 years^[1]^. PD-1 and PD-L1 inhibitors have an objective response rate of around 20%-40% in a variety of metastatic tumor types^[3-6]^, and like CTLA-4, a significant number of patients lose response to antiPD-1 mabs^[7,8]^. Combinations of anti-CTLA4 + antiPD1 ICIs have also been approved by the FDA demonstrating improved response rates compared to ICI monotherapy, but are associated with significant increases in autoimmune adverse effects^[9]^.

## What are immune checkpoints?

Immune checkpoints are cellular synapses that are part of the immune system and under physiological condition prevent autoimmunity and regulate immune systems response to infection. Immune checkpoint dysregulation occurs in cancer as a mechanism that leads to tumor escape from immune surveillance. Activation of T cells requires the interaction of T-cell receptor (TCR) with the complex that includes the major histocompatibility complex (MHC) I or II and cognate antigen present on antigen presenting cells (APC). Engagement of TCR initiates a second interaction between CD28 (on T-cell) with B7 present (on APC), which signals through phosphoinositide-3-Kinase (PI3K) and protein kinase B (AKT) pathway^[10]^. CTLA-4 which is upregulated when T-cells are activated, competitively inhibits the interaction of CD28 with B7 inhibiting T- cell proliferation^[11]^. Downstream inhibition of CD28 mediated PI3K/AKT pathway is another mechanism of T cell suppression^[12]^. CTLA-4 can also inhibit T cell activation by trans-endocytosis of B-7 ligands on the APC and degradation in the CTLA-4 expressing cells^[13]^. In addition, CTLA-4 is essential for the suppressive function of CD4 T regulatory (Treg) cells that is partly mediated by the downregulation of B7 on APC^[14]^.

PD-1 is another checkpoint that is expressed upon T cell activation and is present on tumor infiltrating lymphocytes (TIL). Expression of PD-1 in TIL has been shown to be a marker of T-cell exhaustion with decreased production of effector cytokines^[15]^. PD-L1 is expressed on many immune cells as well as on several human malignancies and is induced when cells are exposed to interferon (IFN)-γ^[16]^. PD-1/PD-L1 interaction can directly inhibit the TCR signaling by dephosphorylation of downstream molecules^[17]^. Like CTLA-4, PD-1 can inhibit CD28 mediated PI3K/AKT pathway signaling^[12]^. PD-1/PD-L1 pathway also regulates Treg development and functioning mediated by the reduced signaling via the AKT-mTOR pathway; a pathway required for the development of Treg cells^[18]^. Tables 1 and 2 summarize the mechanism of resistance to ICI and potential combinations therapies to overcome ICI resistance.

**Table 1:** Mechanism of primary and acquired resistance to checkpoint inhibitor^[99]^

Primary resistance	
Tumor cell	Low mutation burden/ Lack of neo-antigen Lower MHC-I expression Defects in β2M T-cell exclusion program Overexpression of VEGF Activation of PI3K-AKT and WNT/β-catenin pathways Mutations in the IFN-γ pathway. Epigenetic modulation of antigen processing and cytokine production
Tumor microenvironment	Severe Exhaustion of T-cells Increase in number and activity of MDSC in the tumor microenvironment Increase in Tumor associated macrophage Production of metabolic inhibitors like IDO-1 and adenosine in the tumor microenvironment Immunosuppressive cytokines like TGF-β, IL-10
Acquired resistance	Defects in β2M JAK1/2 mutation Presence of alternative checkpoints

MHC: major histocompatibility complex; VEGF: vascular endothelial growth factor; PI3K: phosphoinositide-3-Kinase; AKT: protein kinase B; IFN: interferon; MDSCs: Myeloid-derived suppressor cells; IDO: indoleamine-2,3 dioxygenases; TGF: tumor growth factor; IL: interleukin

**Table 2:** Combination therapy to overcome resistance to checkpoint inhibitor

Mechanism of resistance	Potential combination therapy
Low mutation burden/lack of neo-antigens	ICI + radiation ICI + chemotherapy ICI + oncolytic virus
T-Cell Exclusion program	ICI + CDK4/6 inhibitor
Defects in antigen processing/β2M	ICI + interferon
VEGF production by tumor	ICI + VEGF inhibitor
Epigenetic modulation leading to decrease in tumor antigenicity	ICI + hypo-methylating agent ICI + HDAC inhibitors
Secondary checkpoints	ICI + anti-TIM3 ICI + anti-TIGIT
Immunosuppressive cells in tumor microenvironment	ICI + PI3K-γ selective inhibitor ICI + HDAC inhibitor ICI + anti-CSF1R
Inhibitory molecules and cytokines	ICI + IDO-1 inhibitor ICI + anti-CD73mab ICI + TGF-β receptor kinase

ICI: Immune checkpoint inhibitors; VEGF: vascular endothelial growth factor; HDAC: histone deacetylase; TGF: tumor growth factor

## Mechanisms of resistance

### Qualitative and quantitative aspects of mutational burden and responses

Immune recognition of tumor cells requires tumor expression of non-self-antigens (neo-antigens) or embryonic antigens which can be recognized as foreign by the immune system. These neo-antigens are typically derived from a somatic mutation of existing proteins or from the expression of viral proteins that have been incorporated into the cell DNA as part of carcinogenesis^[19]^. Increased tumor somatic mutation burden (TMB) is common in melanoma, non-small lung cancers and bladder cancer^[20,21]^. Although not exclusive, high TMB has been associated with higher ICI response rates and better clinical outcomes in these tumors^[22,23]^. On the other hand, tumors with low TMB like pancreatic cancer^[20]^ have been associated with an immune desert in their tumor microenvironment (TME) and have shown primary resistance to ICI therapy^[24]^. Several strategies have been attempted with varying degrees of success to switch the TME from an immune desert to a site which promotes immune cell infiltration by either inducing mutations and immunogenic cell death using radiation, chemotherapy or oncolytic viruses^[25-27]^.

Tumor heterogeneity and branched evolutionary drift may also contribute to resistance. While tumor heterogeneity has been well recognized in histopathologic specimens, genetic evolution of tumors is now well documented^[28]^. McGranahan *et al*.^[29]^ have shown that the presence of intra-tumor heterogeneity defined by the presence of subclones, is associated with lower response rates. They proposed that the presence of several subclones could dampen the T cell response due to inadequate antigen dosage leading to a less than optimal response to immunotherapy. Tumor heterogeneity has been recognized as a mechanism of drug resistance in chemotherapy and the concepts of how best to sequence or combine therapies can be applied to ICI by recognizing the mechanisms of resistance and opportunities to overcome these pathways^[30]^.

Even though the mutational burden has been shown to be an important aspect of tumor immunogenicity and response to ICI, not all patients with high TMB respond to ICI, and conversely some low TMB patients will respond to therapy. Snyder *et al*.^[23]^ have shown that although high TMB was associated with longer overall survival with anti-CTLA4 therapy; by itself, TMB was not sufficient to predict clinical response. Rather they proposed the presence of a certain tetrapeptide sequence that is recognized by T-cells as a basis of response to anti-CTLA4 therapy. Hugo *et al*.^[31]^ have shown that response rates to anti-PD1 in melanomas don’t correspond to the mutational burden, although a statistically significant relation was seen between high tumor burden and overall survival. Alternatively, this group demonstrated that a gene signature related to mesenchymal transition, wound healing and angiogenesis was associated with tumors unresponsive to anti-PD1 therapy and worse overall survival. This signature was upregulated in other tumors including a majority of pancreatic adenocarcinomas which are resistant to anti-PD1 based therapy. Similarly, Jerby-Arnon *et al*.^[32]^ have defined a resistance program that predicts T cell exclusion and predicts resistance to immunotherapy. This program was present prior to ICI therapy and enhanced in resistant lesions. The CDK4/6 inhibitor, abemaciclib decreased the expression of this program in melanoma cell lines and showed synergistic action with ICI in murine models. Similar results were also seen in other murine models where the combination of abemaciclib with anti-PD1 not only led to dramatic upregulation of genes related to T cell activation but also suppression of cell-cycle related genes^[33]^.

T-cell engagement of target requires recognition of the cognate antigen in the context of MHC class I molecules. Lower expression of MHC-I was seen in patients with squamous cell lung cancer and no survival advantage was seen even in patients with high tumor burden^[29]^. Similarly, loss of β2M, which stabilizes the MHC class I complex, has also been noticed in melanoma treated with dendritic cell vaccine and could be a possible mechanism of resistance^[34]^. Interferons are known to increase expression of class I MHC, β2M as well as restore deficiencies in antigen processing pathways [Transporter associated with antigen processing (TAP) molecules] and have recently been tested in combination with ICIs. Loss of mutations that are associated with neo-antigen formation has also been seen during ICI therapy although the TMB essentially remained unchanged in these patients^[35]^.

## Lack of T-cell infiltration and suppression of T cell function

Immature dendritic cells (DCs) are involved in phagocytosis of tumor antigen and the processing of tumor antigens. They then mature and migrate to secondary lymphoid organs where they interact with naïve T cell leading to their activation, expansion and later infiltration in the tumor.

Vascular endothelial growth factor (VEGF) is a pro-angiogenic cytokine which is secreted by several solid tumors. It has immunomodulatory actions and has been shown to decrease T-cell proliferation and cytotoxic function^[36]^. Along with this VEGF has been shown to decrease the maturation of DCs^[37]^. Blocking VEGF using monoclonal antibody increased T cell infiltration into the tumor^[38]^. Supporting the role of VEGF inhibitors in ICI resistance is a phase I study of ipilimumab and bevacizumab in patients with metastatic melanoma which showed a disease control rate of 67% and median survival of 25 months, which are appreciably more than historical data with ipilimumab monotherapy^[39]^. Downstream pathways of VEGF receptor have also been implicated in resistance formation. Peng *et al*.^[40]^ have shown that mutated PTEN is associated with inferior response to ICI. PTEN mutated tumors were associated with a decrease in T cell infiltration. PTEN mutation leads to activation of the PI3K-AKT pathway. Combining PI3K inhibitor with ICI showed synergistic anti-tumor activity. The group was also able to show an increase in VEGF production as a possible mechanism, and enhanced T cell infiltration and antitumor activity when combined with anti-VEGF-blocking antibody. One case report of a patient with metastatic uterine leiomyosarcoma who developed a single ICI resistant metastasis had biallelic PTEN mutation and increased expression of VEGFA which supports this resistance pathway^[41]^.

The activation of the WNT/β-catenin pathway has also been shown to be involved with immune evasion. Wnt5a production from melanoma cells also seem to upregulate the β-catenin pathway in the DCs in the tumor microenvironment leading to differentiation of naive T cell to Treg cell^[42]^. Activation of the pathway also reduces infiltration and activation of CD103+ DCs due to reduced expression of CCL4 leading to decrease in T cell infiltration^[43]^. Intra-tumoral injection of dendritic cell restored the responsiveness of tumor to ICI^[43]^.

Other important pathways for resistance include STK11/LKB1 mutation in KRAS mutant lung adenocarcinoma that has been shown to directly induce resistance to ICI therapy, with a significant decrease in the response rate and overall survival^[44]^. Another resistance pathway is acquired changes in JAK-STAT. Zaretsky *et al*.^[45]^ have shown that mutation in Janus Kinase-2 (JAK-2) is a mechanism of resistance to anti-PD1 therapy. They showed that mutation of JAK-2 in melanoma cells leads to a lack of response to IFN-γ which includes lack of phosphorylation of STAT-1 and STAT-3 and decreased expression of MHC-1, PD-L1 and TAP-1. Significantly high frequency of mutations in the IFN-γ pathway was also seen in patients who were non-responders to ipilimumab compared to patients who responded to therapy^[46]^.

Epigenetic modulation has also been implicated in resistance to ICI. Epigenetic modulation has been shown to decrease the expression of tumor-associated antigen through alteration of the antigen processing machinery, downregulation of MHC-I and the decreased expression of MHC-I on the cell surface^[47]^. Treatment of renal cancer cells with DNA hypomethylating agent induces the expression of cancer testes antigens, which are normally not expressed by these cells^[48]^. In melanoma cell lines these agents increase the expression of MHC-I and increase gp100 specific CTL mediated lysis^[49]^. Similarly, when mice and human melanoma cell lines were treated with histone deacetylase inhibitor panobinostat, an increase in the expression of MHC-I along with other costimulatory molecules was seen^[50]^. Increased expression of melanoma differentiation antigens was also seen in these cell lines^[50]^. Peng *et al*.^[51]^ have also shown in mouse ovarian cell line models that DNA methylation decreases the production of tumor-mediated Th1 type cytokines including CXCL9 and CXCL10, leading to decreased T cell infiltration into the tumor. Treatment with epigenetic modulator was associated increase in the expression of these cytokines and had synergistic activity in tumor control when combined with T-cells. Several other clinical trials combining epigenetic modifiers and ICI are ongoing^[52]^. A phase 2 study of the combination of nivolumab with azacitidine (a hypo-methylating agent) in patients with relapsed/refractory acute myeloid leukemia showed an overall response rate(ORR) of 33% which was higher than historical ORR with azacitidine alone^[53]^.

## Presence of alternative checkpoints and severe T cell exhaustion

Several other checkpoints apart for PD-1 and CTLA-4 have been discovered which contribute to tumor resistance to available ICI therapy. T-cell immunoglobulin mucin (TIM)-3 is found on Th1 and cytotoxic T cell and act as a negative regulator of their function^[54]^. It also forms a part of the exhausted T cell phenotype which is associated with a lack of proliferation and cytotoxic activity^[54]^. Like TIM-3, lymphocyte activating gene (LAG)-3 is another checkpoint which is expressed on exhausted T cells^[55]^. B- and T-lymphocyte attenuator (BLTA) is a checkpoint that has been shown to be a late marker of exhaustion. CD8+BLTA+ T cells showed high co-expression of other inhibitory receptors compared to CD8+ cells with other inhibitory receptors^[56]^. Koyama *et al*.^[57]^ showed an increase in the surface levels of TIM-3 and LAG-3 on CD8 cells in murine models of lung cancer resistant to anti-PD1 therapy when compared to untreated tumors. Addition of antibody to TIM-3 after the development of anti-PD1 resistance lead to an increase in overall survival of mice. An increase in overall survival was seen when anti-PD1 was added to anti-TIM-3 therapy in murine models of glioblastoma^[58]^. Similarly, T-cell immunoglobulin and ITIM domain (TIGIT) is another checkpoint which is expressed on CD8+ T-cells, NK cells and Treg^[59]^. *In-vitro* PD-1 blockade causes upregulation of TIGIT in tumor antigen-specific CD8+ T-cells, a potential mechanism of resistance. Dual blockade of TIGIT and PD-1 increased IFN-γ, TNF and T cell proliferation^[59]^. V-domain Ig suppressor of T-cell activation (VISTA) belongs to the immunoglobulin superfamily and is another immune checkpoint with potent negative regulation of T-cell function. VISTA is expressed at high levels on monocytes, granulocytes, and macrophages, and at lower densities on T-cell populations within the tumor microenvironment. Kuklinski *et al*.^[60]^ found that expression of VISTA in primary melanomas was associated with a significantly worse disease-specific survival, and is regulated differently than PD1 and represents another potential pathway of resistance to established ICIs.

Blackburn *et al.*^[61]^ reported two subsets of CD8+ cells in mice chronically infected with lymphocytic choriomeningitis virus based on expression of PD-1. The cells with intermediate PD-1 expression were rescuable with blockade of PD-1/PD-L1 pathway with more expansion and protective immunity, and reduced apoptosis when compared to cells with high PD-1 expression which were not rescued by using anti-PD-L1. CD8+ T cells with high PD-1 expression (PD-1 high) also express other inhibitory checkpoints associated with T cell exhaustion and the expression of multiple inhibitory checkpoints correlates inversely with T cell function as evident by lower staining for intracellular IFN-γ and TNF^[62]^. Different stages of T cell exhaustion have been described, starting with the loss of production of IL-2 followed by TNF and ultimately IFN^[63]^. Presence of partially exhausted CD8+ T cells capable of producing IFN but not IL-2 or TNF correlated with response to anti-PD1 therapy in patients with melanoma^[64]^. The development of an exhausted and dysfunctional state has been linked to epigenetic modulation in genes involved in effector function and memory T cell differentiation^[65,66]^.

## Immunosuppressive tumor microenvironment

Myeloid-derived suppressor cells (MDSCs) alter the function of CD8+ T cells^[67]^. Several mechanisms have been proposed including a decrease in arginine and cysteine, production of free radicals which eventually inhibit the functioning of TCR and IL-2 signaling, decreased trafficking of T cells into the lymph node and tumor, induction of T cell apoptosis and expansion of Tregs^[67]^. Presence of higher baseline numbers of MDSCs in peripheral blood has been seen in non-responders when compared with responders and was also associated with worse overall survival in patients with metastatic melanoma who were on ipilimumab^[68]^. In addition in non-responders, the immunosuppressive activity of MDSCs has been shown to be significantly higher when compared to responders while getting treatment with ipilimumab^[69]^. Similarly, in patients treated with nivolumab after progression on ipilimumab higher baseline MDSC number correlated with progression and poor overall survival^[70]^. Highfil *et al*.^[71]^ have shown in a murine model of rhabdomyosarcoma that CXCL-1 and CXCL-2 are responsible for the trafficking of MDSCs into the tumor. There was a significant tumor shrinkage in the tumor with the use of anti-PD1 in mice reconstituted with CXCR-2 (-/-) hematopoietic cells when compared to wild type mice. De Henau *et al*.^[72]^ showed that inhibition of PI3K-γ decreased myeloid cell migration into the tumor. Combination of ICI and PI3K-γ selective inhibitor significantly delayed tumor growth in previously ICI resistant tumors. Combination of HDAC inhibitor entinostat and ICI has also been shown to have synergistic effects, capable of sensitizing tumors which are resistant to ICI. This appears to be mediated by a decrease in the immunosuppressive potential of MDSC^[73]^. Blood and tissue MDSCs also correlate with several clinicopathologic factors and may predict for pathological complete response providing further evidence that MDSCs play an important role across tumor types^[74]^.

Tumor-associated macrophages (TAM) are associated with poor survival in several malignancies^[75-77]^. DeNardo *et al.*^[77]^ showed an inverse relation between infiltration of CD68+ macrophages and CD8+ T-cells in a breast cancer model. They also proposed that colony stimulating factor(CSF)-1 is responsible for macrophage infiltration, and its blockade is responsible for the decrease in TAM, increase in CD8+ cells tumor infiltration, increase in expression of cytotoxic effector molecules and decrease in the expression of arginase-1. Similar results have been seen in other tumor models too^[78,79]^. CFS-1/CSF-1R signal blockade modestly reduces T cell growth when used as a single agent due to upregulation of T cell checkpoint. Combination of ICI and CSF1R blockade, on the other hand showed impressive tumor control in murine models of pancreatic carcinoma, which is generally resistant to ICI^[80]^.

Indoleamine-2,3 dioxygenases (IDO) is an IFN-γ induced intracellular enzyme which is involved in tryptophan metabolism^[81]^. It is expressed by several human tumor cell lines and activated DCs and is shown to decrease T cell proliferation^[81-83]^. IDO causes inhibition of T cell proliferation by decreasing tryptophan which is an essential amino acid and production of metabolites which have immunosuppressive activity^[81,84]^. Holmgaard *et al*.^[85]^ showed that in murine models of melanoma combination of anti-CTLA4 with anti-IDO resulted in a significant decrease in tumor sizes and improved overall survival. Monotherapy with either agent didn’t have any significant effects. Similarly, in a murine model of glioblastoma combination of anti-PD-L1, anti-CTLA, and IDO inhibitor lead to a durable response in all animals^[86]^. Unfortunately, the phase III study of pembrolizumab +/- epacadostat did not find any benefit from the addition of the IDO1 inhibitor^[87]^. On the other hand, an earlier study of an IDO1 inhibitor with ipilimumab in patients with metastatic melanoma has been reported that showed good tolerance and disease control rate of 75%^[88]^, but will need to be followed by a well-designed phase III study.

Adenosine which is hydrolyzed from AMP by the extracellular domain of CD73 present on tumor cells has also been implicated in apoptosis of T cells and suppression of their activation and effector function^[89]^.The combination of anti-PD1 or anti-CTLA-4 mab with anti-CD73mab has been shown to have impressive therapeutic activity and increases survival in a murine model. This was associated with an increase in antigen-specific CD8+ TILs, increase in Th1 related genes, and a decrease in expression of CD73 on tumor and TILs^[90]^. Tumor growth factor (TGF)-β is another cytokine that is produced by the tumor and the microenvironment. In normal tissues, it prevents tumorigenesis and maintains homeostasis although in cancerous tissue it promotes tumor progression and escape from immune surveillance^[91]^. Thymoma murine model which secretes TGF-β has been shown to decrease the cytotoxic activity of CD8+ T cells^[92]^. Combination of anti-CTLA-4 with TGF-β receptor kinase inhibitor in melanoma murine model showed synergistic activity with an increase in the CD8+/Treg ratio^[93]^. Phase I trials of TGF-β inhibitors as monotherapy and in combination with ICI are currently ongoing^[94]^. Immune suppressive factors in the tumor microenvironment TME have also been suggested as pathways of resistance. Tumor-derived immunosuppressive cytokines, like IL-10 and exosomes, have been noted^[95,96]^. These observations will lead to novel combinatorial therapies directed at these pathways.

Prior treatment may also influence changes in tumor microenvironment causing resistance to ICI. Hugo *et al*.^[97]^ have shown that in melanoma which develops resistance to MAPKi there is positive enrichment in genes related to inflammation (likely mediated by macrophages) and monocyte. There was a decrease in CD8 T cells (overall and melanoma-tumor reactive) along with downregulation of genes involved in antigen processing and presentation. This was corroborated in a retrospective study which showed that in patients who discontinued BRAFi with/without MEKi, there were no objective responses with ipilimumab^[98]^.

## Conclusion

Although ICIs have had a major impact on the therapy of many tumor types and subsequent patients’ lives, the observation of primary and acquired resistance to anti-PD1/PD-L1 and anti-CTLA4 agents leave much room for improvement. Understanding the biology of tumor-associated immune responses and the multiple pathways of T cell inhibition and exhaustion will provide opportunities to enhance the therapeutic window.

## Declarations

### Copyright

© The Author(s) 2019.
